# Letter to the editor

**DOI:** 10.1007/s00590-012-1004-1

**Published:** 2012-05-15

**Authors:** P. A. van Luijt, I. B. Schipper, P. W. van Egmond

**Affiliations:** Leiden University Medical Centre, Leiden, The Netherlands

Dear Sir

We would like to congratulate Dr. Singer with his excellent results using closed reduction and cast immobilization under general anesthesia for displaced forearm fractures in children.

These exceptional results are, however, not representative of treatment for displaced forearm fractures in children in daily practice. As Dr. Singer himself states in his Letter to the Editor, our results showing that 43.7 % of the children with a dislocated forearm fracture treated with reduction without internal fixation needed a secondary procedure under general anesthesia, are in concordance with those presented in previous publications.

In our patient group, not all patients with a displaced forearm fracture were reduced under general anesthesia. The less displaced and potentially stable fracture patients were often reduced under local anesthesia and therefore excluded from our study group. The differences in results may therefore in part be due to patient selection.

In our discussion, we did emphasize the importance of a correctly molded cast, and in fact, a well-molded cast with three-point fixation was the primary treatment aim for all patients.

The published data, however, show the realistic results of our daily practice in a retrospective analysis. These results suggest that a significant improvement can be accomplished by minimal invasive intramedullary fixation in case of fracture reduction under general anesthesia. We strongly feel that this conclusion holds true for most hospitals treating dislocated forearm fractures in children.

Leiden, April 24, 2012

